# Disease control in oilseed rape: Current insights and perspectives for next‐generation integrated pest management

**DOI:** 10.1002/ps.71014

**Published:** 2026-06-19

**Authors:** Jon S West, Małgorzata Jędryczka, Nazanin Zamani‐Noor

**Affiliations:** ^1^ Rothamsted Research Harpenden UK; ^2^ Pathogen Genetics and Plant Resistance Team, Institute of Plant Genetics, Polish Academy of Sciences Poznan Poland; ^3^ Julius Kühn‐Institute (JKI), Institute for Plant Protection in Field Crops and Grassland Braunschweig Germany

**Keywords:** biocontrol, chemical fungicides, cultural practices, decision support, genetic resistance

## Abstract

Winter oilseed rape (WOSR; *Brassica napus*) is a key crop in Europe; yet, diverse pathogens, including *Pythium* spp., *Rhizoctonia solani, Plasmodiophora brassicae, Pyrenopeziza brassicae, Leptosphaeria maculans* (*Plenodomus lingam*), *Leptosphaeria biglobosa* (*Plenodomus biglobosus*), *Erysiphe cruciferarum, Sclerotinia sclerotiorum, Alternaria brassicae, Alternaria brassicicola*, and *Verticillium longisporum* frequently threaten its productivity. Integrated pest management (IPM) provides a sustainable framework for minimizing disease impact while reducing reliance on chemical fungicides. This review introduces current knowledge on IPM strategies for control of diseases in European WOSR, including crop rotation and residue management, tillage practices, sowing dates and plant density, host resistance and breeding strategies, and targeted fungicide applications guided by forecasting models that limit pathogen impact. Recent advances in decision support systems, remote sensing, and predictive disease modelling are highlighted, illustrating their potential to enhance precision and efficiency in disease management. We also discuss challenges such as climate variability, evolving pathogen populations, and policy and regulatory considerations, as well as future perspectives for integrating novel biological control agents, digital and biotech tools into IPM frameworks. Overall, this review provides a comprehensive overview of knowledge and emerging approaches, aiming to inform researchers and advisors on sustainable strategies for managing major WOSR diseases in Europe. © 2026 The Author(s). *Pest Management Science* published by John Wiley & Sons Ltd on behalf of Society of Chemical Industry.

## Graphical abstract

Winter oilseed rape in Europe suffers frequent pathogen losses. This review outlines key integrated pest management (IPM) strategies, rotation, residue and tillage management, optimized sowing, resistance, and targeted fungicides supported by forecasting. It highlights advances in decision tools, challenges from climate and regulation, and emerging biological, digital, and biotech solutions for sustainable management.

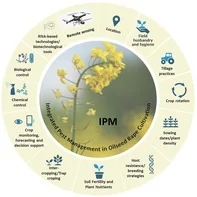

## 1 INTRODUCTION

### 1.1 Overview of winter oilseed rape cultivation and importance

Winter oilseed rape (WOSR; *Brassica napus* L.; genome AACC, 2*n* = 38), also known as canola in North America and Australia, originated from spontaneous interspecific hybridization between turnip rape (*Brassica rapa* L., syn. *campestris*; genome AA, 2*n* = 20) and cabbage (*Brassica oleracea* L.; genome CC, 2*n* = 18), leading to the formation of an amphidiploid species.^1^, ^2^ Domesticated as an oilseed crop approximately 400–500 years ago, intensive breeding efforts over the past four decades has propelled it into a major global crop.^3^ Oilseed rape (OSR) is widely cultivated for its oil‐ and protein‐rich seeds, which serve diverse applications in the food, feed, and industrial sectors.^4^ The adaptability of OSR to various climates, combined with its high yielding potential and positive impact on soil health, makes it an excellent choice for sustainable farming.^3^, ^4^, ^5^ Additionally, incorporating OSR into crop rotations with cereals or legumes, aids in managing pests and diseases, disrupting weed cycles, and improving soil fertility, all essential components of sustainable agriculture.^6^, ^7^, ^8^ Moreover, cultivating OSR promotes biodiversity by creating habitats for beneficial organisms, as well as providing essential resources for pollinators.^9^, ^10^ In Europe, WOSR is widely cultivated, as a profitable break‐crop particularly in France, Germany, Poland, Romania, the Czech Republic, and the United Kingdom (UK) (Fig. 1). It is predominantly grown as a winter crop and thrives in the temperate regions of central and western Europe, where conditions favour optimal seed and oil yields.^3^ In these areas, the average annual yield of WOSR typically ranges from 3.3 to 4.3 metric tons per hectare.^3^

**Figure 1:**
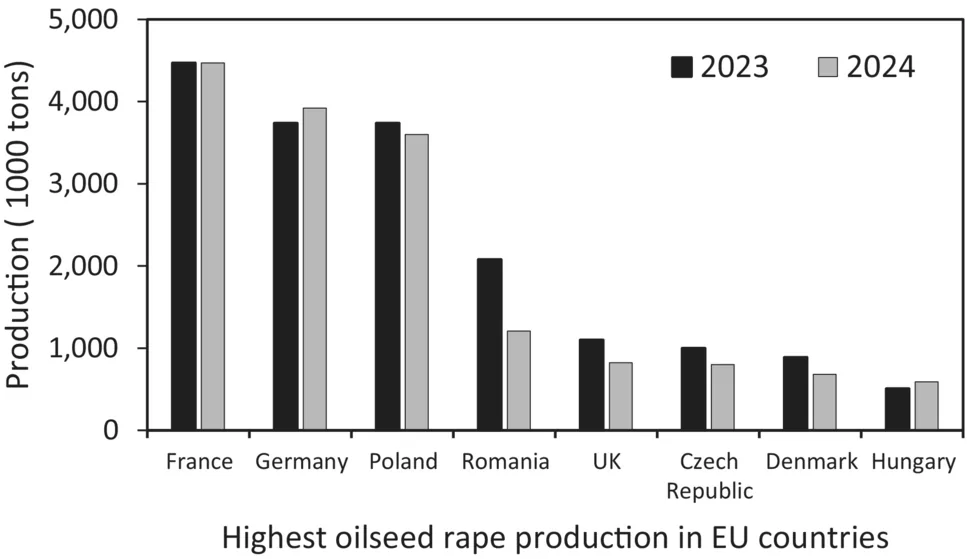
Winter oilseed rape production (thousand metric tons) in major European countries. European Commission, Agricultural Market Information and UK Department for Food and Rural Affairs statistics.

WOSR cultivation faces growing economic losses caused by pests, diseases and climate change, intensified by increasing pesticide resistance. These challenges highlight the critical importance of developing advanced breeding programmes and integrated pest management (IPM).^11^, ^12^ Such forward‐looking strategies are essential to ensure the future sustainability and profitability of WOSR cultivation.

### 1.2 Significance of pathogens in winter oilseed rape

WOSR cultivation is frequently affected by various diseases caused by diverse pathogens, including fungi, oomycetes, protists, bacteria, and viruses. These disease pressures are intensified by changing climatic conditions, evolving pathogen populations, and the limited availability of effective control measures.^13^, ^14^ Additionally, the intensification of WOSR production and the declining genetic diversity of cultivars have increased the crop's susceptibility to several diseases. Among the various diseases threatening WOSR cultivation, several caused by fungi, oomycetes, and protists (Fig. 2) are particularly damaging economically and will be the focus of this review. These pathogens can lead to substantial yield losses, reduced seed quality and quantity, and increased production costs, which can even restrict OSR cultivation in certain regions and limit crop rotation options.^13^, ^15^, ^16^, ^17^, ^18^, ^19^, ^20^, ^21^, ^22^, ^23^, ^24^

**Figure 2:**
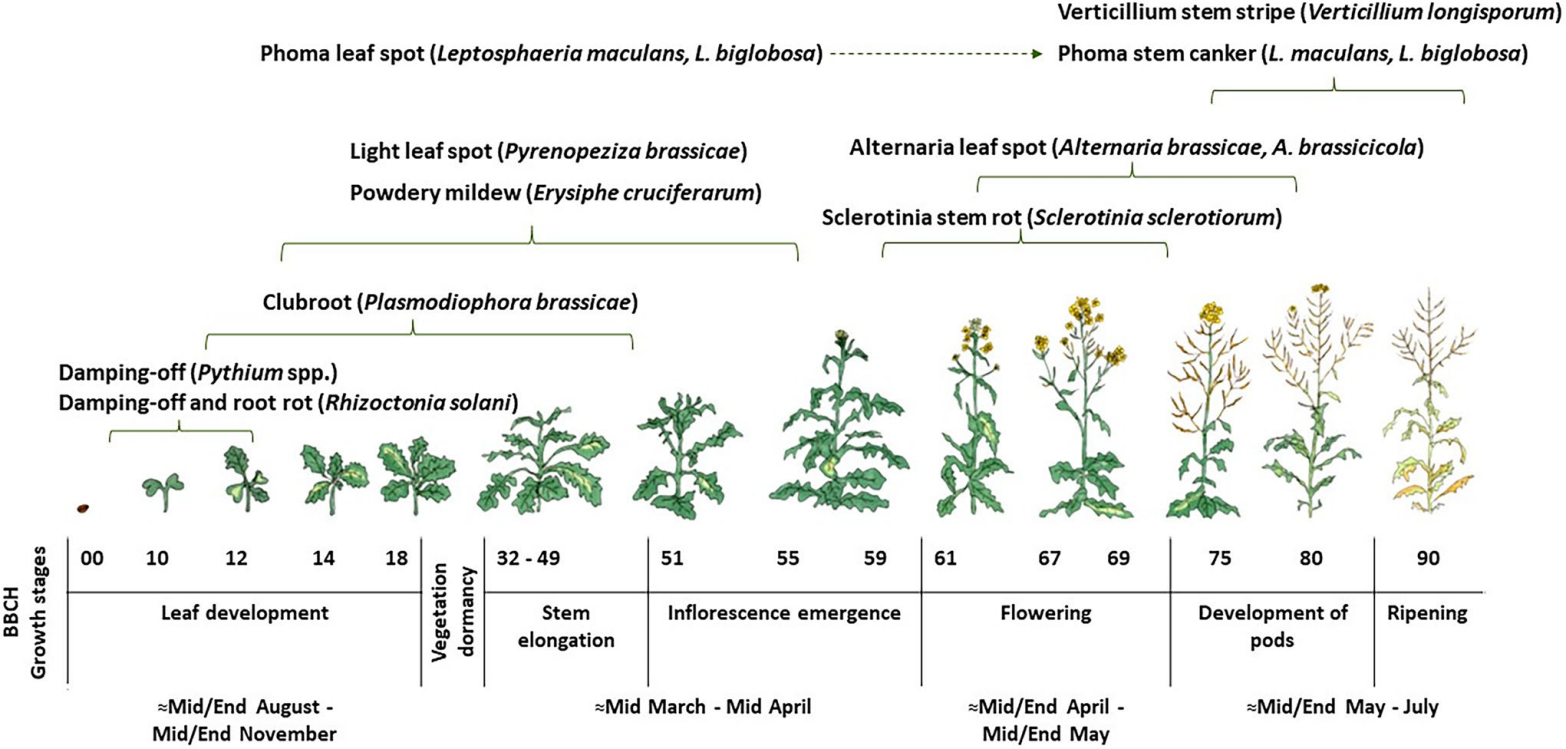
Overview of the major fungal, oomycete, and protist diseases affecting winter oilseed rape in Europe, illustrating the typical timepoints of their occurrence during the growth cycle of winter oilseed rape.

Figure 3 shows the typical symptoms associated with these diseases of WOSR, illustrating the wide range of infection sites and tissue damage they cause.

**Figure 3:**
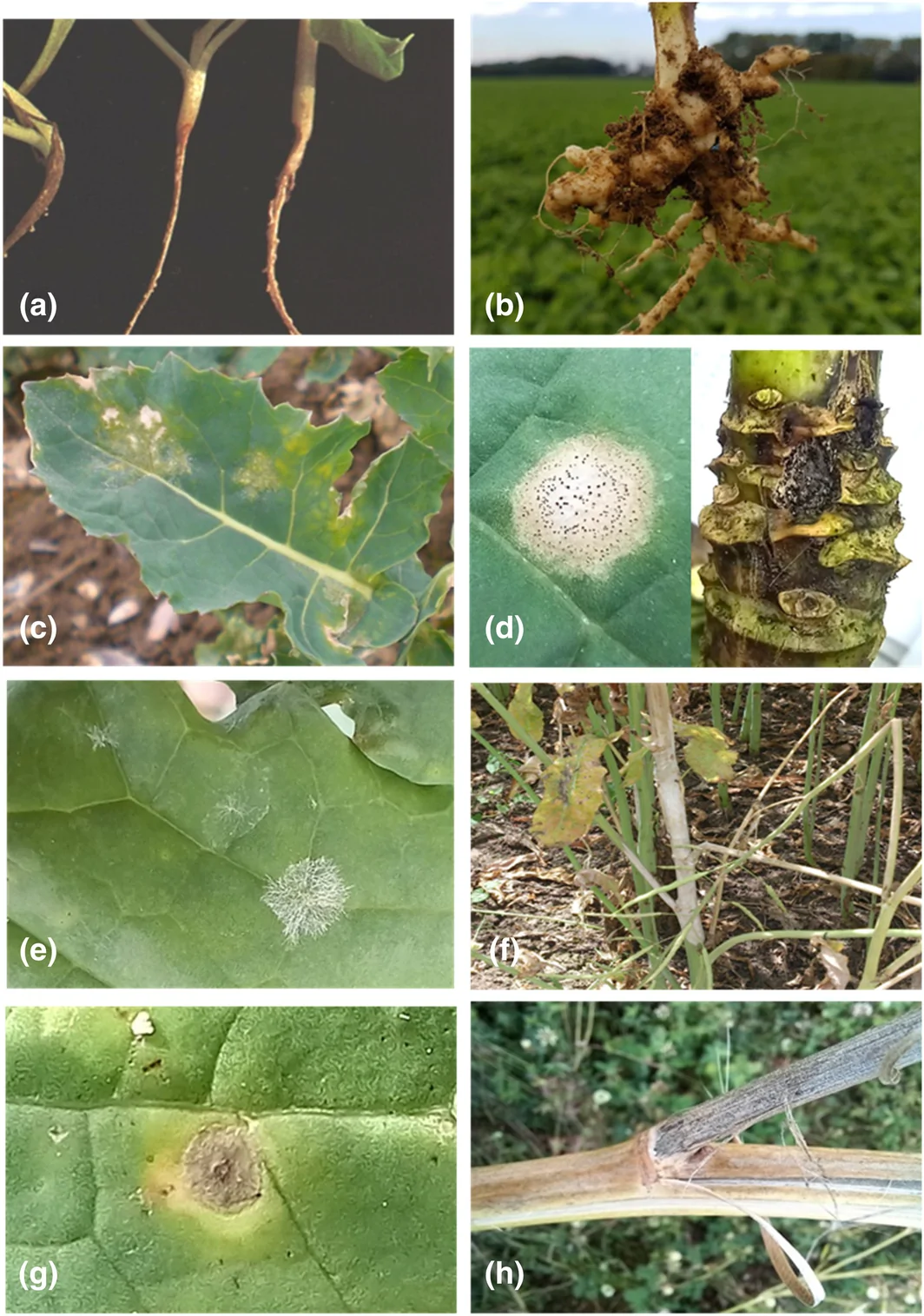
Typical symptoms of the major fungal, oomycete, and protist diseases impacting winter oilseed rape under European field conditions. (a) Damping‐off (*Pythium* spp.); (b) clubroot (*Plasmodiophora brassicae*); (c) light leaf spot (*Pyrenopeziza brassicae*); (d) phoma leaf spot/stem canker (*Leptosphaeria maculans*, *Leptosphaeria biglobosa*); (e) powdery mildew (*Erysiphe cruciferarum*); (f) sclerotinia stem rot (*Sclerotinia sclerotiorum*); (g) Alternaria leaf spot (*Alternaria brassicae*, *Alternaria brassicicola*); (h) Verticillium stem stripe (*Verticillium longisporum*).

### 1.3 Principles of integrated pest management (IPM)

IPM is a broad approach to sustainable farming, aiming to prevent, detect and control pests (pests, weeds and diseases) using a coordinated system of long‐term and reactive strategies that integrate traditional cultural methods with physical, biological and where necessary, chemical control to facilitate cost‐effective crop production. The foundations of IPM are traditional and preventive cultural practices, including diverse crop rotation, the use of pest and disease‐resistant varieties, and cultural practices to harness nature‐based solutions such as conserving beneficial organisms and further diversification through cultivar mixtures, under‐planting or inter‐cropping. This is augmented by other key components including: surveillance, forecasting and monitoring tools, which are used to optimize interventions by the farmer that comprise physical and mechanical control, biological control or chemical control.^25^ These aspects are discussed fully in later sections of this review. IPM has been adopted widely in the production of arable crops because it is cost effective by harnessing nature wherever practical and avoiding unnecessary, costly applications of crop protection products (whether pesticides or biologicals). While IPM has been enhanced by precision‐farming practices and uptake of sophisticated sensor technologies in horticulture and other high‐value crops, arable crop production has recently moved more towards a slightly lower‐input and more regenerative version of IPM in response to substantial increases in fertilizer prices. This has increased reliance on host resistance and choice of varieties that yield reasonably well under little or no pesticide applications.

## 2 MAJOR AND IMPORTANT DISEASES OF OILSEED RAPE

WOSR is affected by several economically significant diseases caused by fungi, oomycetes, and protists.^26^ These include damping‐off and root rot, powdery mildew, clubroot, sclerotinia stem rot, phoma stem canker, light leaf spot, Alternaria leaf and pod spot, and Verticillium stem striping (Fig. 3). The causal agents for these diseases are given in Table 1. Disease occurrence and severity varies across Europe depending on climatic conditions, cropping practices, and varietal susceptibility.^27^ An overview of the major and economically important diseases, their geographical distribution, favourable conditions, and the main IPM strategies applied in Europe is summarized in Table 1.

**Table 1:** Economically important fungal, oomycete, and protist diseases of winter oilseed rape in Europe and their main integrated pest management (IPM) strategies

Disease name/pathogen	Geographical distribution in Europe	Favourable conditions	Main IPM strategies	Selected references
Damping‐off/*Pythium* spp.	Occurs sporadically across Europe	High soil moisture, low temperatures at seedling stage	Seed treatment with fungicides, well‐drained seedbeds, appropriate sowing depth	28, 29
Damping‐off and root rot/*Rhizoctonia solani*	Widespread in Europe	Warm, moist soils; poor seedbed preparation	Seed treatments with fungicides, crop rotation, good soil structure	30
Clubroot/*Plasmodiophora brassicae*	Widespread across Europe; Germany, Poland, Sweden, UK, Austria, Czech Republic	Acidic soils, poor drainage	Raise soil pH > 7, resistant cultivars, sanitation, long crop rotation	18, 31, 32, 33
Light leaf spot/*Pyrenopeziza brassicae*	Primarily UK and Ireland; emerging in Germany, Poland and France	Cool, wet autumn/spring	Resistant cultivars, regular monitoring, foliar fungicide treatments, early warning systems	34, 35, 36, 37
Phoma leaf spot, stem canker/*Leptosphaeria maculans, Leptosphaeria biglobosa*	Western and central Europe: UK, France, Germany, The Netherlands	Mild, wet autumns and winters	Resistant cultivars, stubble management, crop rotation, foliar fungicide treatments	38, 39, 40, 41
Powdery mildew/*Erysiphe cruciferarum*	Scattered outbreaks in central, western, and southern Europe; more common in drier regions	Warm, dry weather with high humidity in the canopy; dense stands with poor air circulation	Use of resistant/tolerant cultivars (where available), moderate plant density, crop rotation, fungicide use if thresholds are exceeded	42, 43
Sclerotinia stem rot/*Sclerotinia sclerotiorum*	Widespread across Europe; higher incidence in France, Germany, UK, Poland	Warm, moist weather during flowering	Crop rotation, biocontrol (*Coniothyrium minitans*), foliar fungicides treatments at flowering, early warning systems	19, 44, 45
Alternaria leaf spot/*Alternaria brassicae, Alternaria brassicicola*	Southern and eastern Europe; sporadic in the North	Warm, humid conditions	Foliar fungicides treatments	46, 47
Verticillium stem stripe/*Verticillium longisporum*	Germany, Sweden, UK, Poland; increasing incidence in France	Dry soils, continuous oilseed rape cropping	Crop rotation with non‐hosts, soil health, resistant lines in breeding	48, 49

*Note:* The pathogens in the table are organized in the order of their typical appearance during the growing season.

Current advances in genetics and genomics have significantly improved our understanding of the genomes of all major pathogens affecting WOSR. These developments have enabled detailed insights into pathogenicity, host–pathogen interactions, and population diversity. A summary of the key genomic resources available for these pathogens is provided in Table 2.

**Table 2:** Overview of sequenced genomes of important fungal, oomycete, and protist winter oilseed rape (WOSR) pathogens

Number	Pathogen	Genome identifier	Exemplary isolate	Genome size	Number of chromosomes	Number of operational taxonomic units	Number of genes (proteins)
1	*Pythium* spp.	GCA_000143045.1	DAOM BR144	42.8 Mb	18	4 species	~15290
2	*Rhizoctonia solani*	GCF_016906535.1	7/3/14	36.9 Mb	16	14 groups	~12400
3	*Plasmodiophora brassicae*	GCA_001049375.1	e3	24.2 Mb	20	~42 pathotypes	~9730
4	*Pyrenopeziza brassicae*	GCA_958299125.1	15WOSR64‐SS1	43.8 Mb	N/A	1	~11700
5	*Leptosphaeria maculans*	GCF_000230375.1	v23.1.3	45.1 Mb	18	2 types + high genotypic diversity	~12500
6	*Leptosphaeria biglobosa*	GCA_900465125.1	G12‐1	29.5 Mb	1‐6‐19	2 types	~10000
7	*Erysiphe cruciferarum*	GCA_000208805.1	UEA‐Wa‐1	85.8 Mb	11	1	~7000
8	*Sclerotinia sclerotiorum*	GCF_000146945.2	1980 UF‐70	38.3 Mb	16	1	~14500
9	*Alternaria brassicae*	GCA_004936725.1	J3	30.6 Mb	17	1	~10650
10	*Verticillium longisporum*	GCA_019188255.1	Vl‐s1	72.5 Mb	15–17	3 hybrid types	~20200

*Note:* NCBI database accessed on 16 June 2026.

## 3 CULTURAL PRACTICES IN DISEASE MANAGEMENT

### 3.1 Crop rotation and residue management

Over the past few decades, economic incentives have encouraged farmers to expand OSR cultivation, resulting in a substantial increase in global production. This intensification has often been accompanied by shorter crop rotations compared to historical practices, in some cases to be as frequent as every alternate year, while no more than 1 year in 4 years is recommended. Table 3 presents typical OSR rotations in Europe, including intervals between successive crops. However, intensification has led to the accumulation of OSR‐specific pests and pathogens. Crop rotation functions by spatially or temporally separating susceptible crops from sources of inoculum, especially where inoculum is produced on the crop debris. Long rotations of 4–7 years with non‐host crops are highly effective in reducing soil inoculum of *Plasmodiophora brassicae* ^50^ while rotations of at least 3–4 years, preferably longer, can lower soil sclerotia levels and reduce the risk of *Sclerotinia sclerotiorum* infection.^44^ To be effective, other crops susceptible to sclerotinia should be avoided, such as carrots, beans and potatoes.^51^ *Leptosphaeria maculans* (phoma stem canker) can also be partly managed through crop rotation and residue management,^52^ whereas *Verticillium longisporum* requires a particularly long rotation (7+ years) due to the long‐term persistence of microsclerotia in soil. Aubertot *et al*.^53^ introduced the idea of an extension to standard crop rotation by adding the rotation of varieties with different resistance genes, which was shown to minimize phoma stem canker by sowing varieties that are immune to infection by spores produced nearby on the previous crop debris.^52^ Diseases such as Alternaria leaf spot, downy mildew, and powdery mildew are primarily airborne or seed‐borne and are therefore only moderately influenced by rotation.^18^, ^54^, ^55^, ^56^, ^57^ Nevertheless, many of these pathogens can persist locally due to the widespread availability of inoculum from infected crop residues, volunteer OSR plants, and closely related Brassicaceous weeds, which can serve as alternative hosts and contribute to disease carry‐over between cropping cycles.^50^ While crop rotation is critical for managing OSR diseases, it is equally important to address the role of volunteer OSR plants and Brassicaceous weeds. These volunteers not only serve as alternative hosts for pathogens but also help diseases persist locally, contributing to inoculum carry‐over between cropping cycles. Therefore, effective disease management in subsequent crops relies not only on rotating crops but also on rigorous volunteer and weed management practices.^50^

**Table 3:** Common crop rotations involving winter oilseed rape along with crop‐specific intervals in European countries

Rotation type	Crop sequence	Interval for winter oilseed rape
Intensive (not recommended)	WOSR → winter wheat → WOSR → winter wheat	1 year
Classic three‐crop	WOSR → winter wheat → winter barley	2 years
Diversified with sugar beet	WOSR → winter wheat → sugar beet → winter barley or oats	3 years
Cereal‐dominated	WOSR → winter wheat → winter triticale → winter barley	3–4 years
Extended four‐crop	WOSR → winter wheat → grain legume (peas, beans) → winter barley or oats	3–4 years
Diversified with potatoes	WOSR → winter wheat → potatoes → spring barley	3–4 years
Diversified with cover crops	WOSR → winter wheat → summer crop (maize, sunflower) → cover crop → winter barley or rye	4–5 years

Various policies have promoted cover crops, flower‐strips and intercrops in sustainable agriculture to enhance the ecological value of agricultural landscapes and provide other ecosystem services including enhanced soil health, erosion control, improved water retention, and biodiversity support for pollinators, but they can also harbour pathogens like *Plasmodiophora brassicae* and *Sclerotinia sclerotiorum*. Brassica species used in cover crops or in field margins may act as a reservoir of many diseases if inoculum of those diseases is compatible with OSR. Such pathogen persistence on non‐crop hosts may elevate primary inoculum levels, leading to potential infections in crops.^22^, ^58^ Therefore, effective crop rotation requires strategic selection of both primary crops and complementary cover crops/intercrops, coupled with precise scheduling and integrated soil management.

### 3.2 Tillage practices

Tillage practices play an important role in the epidemiology of OSR diseases by affecting soil structure, residue decomposition, pathogen survival, and microclimatic conditions.^59^, ^60^ Conservation tillage and no‐till systems, while beneficial for soil health and erosion control, can increase the persistence of crop residues on the soil surface, thereby favouring survival and inoculum build‐up of residue‐borne pathogens such as *Sclerotinia sclerotiorum*, *Leptosphaeria maculans* and *Verticillium longisporum*.^61^, ^62^, ^63^, ^64^ In contrast, conventional ploughing tends to bury infected residues, thereby accelerating decomposition and reducing surface inoculum availability, which in turn lowers disease pressure in subsequent crops.^65^ For instance, reduced tillage has been associated with a higher incidence and severity of phoma stem canker due to the prolonged survival of infested stubble on the soil surface.^66^ Similarly, the survival of sclerotia of the *Sclerotinia* spp. is enhanced under reduced tillage conditions, increasing the risk of stem rot outbreaks, particularly when susceptible crops are grown in short rotations.^19^ However, tillage may have a limited impact on soilborne pathogens such as *Plasmodiophora brassicae* or *Rhizoctonia solani*, where inoculum resides deeper in the soil profile or spreads through contaminated equipment and water movement.^18^, ^67^ Therefore, the integration of tillage practices into an effective IPM strategy should consider both agronomic goals and disease risk. Although non‐tillage is favoured by entomologists as a means to enhance parasitoids and predators of invertebrate pests, strategic use of ploughing or occasional deep tillage, especially following high‐disease years, can be an effective measure to reduce the inoculum potential of specific pathogens.

### 3.3 Sowing dates and plant density

Sowing date and plant density are key agronomic factors influencing the onset, development, and severity of OSR diseases.^32^, ^53^ These factors alter crop canopy structure, plant growth dynamics, and the microclimate within the stand, thereby creating conditions that can either suppress or promote disease development. Several studies have demonstrated that adjusting sowing dates and plant density can be integrated effectively into IPM strategies.^31^, ^32^, ^68^, ^69^, ^70^ However, optimizing sowing time and seeding rate should be considered alongside cultivar resistance, crop rotation, and fungicide use to minimize the risk of major fungal diseases in OSR under diverse European growing conditions. Table 4 summarizes the effects of sowing date and plant density on the incidence and severity of major WOSR diseases. It also provides corresponding IPM recommendations to help minimize disease risks through targeted cultural practices. Some very subtle and contrasting effects of sowing density can occur, for example a dense canopy can promote powdery mildew due to high humidity, but the early shedding of shaded lower leaves in the canopy compared to long retention of leaves that remain on plants in good light on sparse canopies can promote disease escape in phoma stem canker due to leaves in the dense canopy being shed before the pathogen has grown down the petiole to reach the stem.

**Table 4:** Effects of sowing date and plant density on major diseases of winter oilseed rape and related integrated pest management (IPM) recommendations

Disease	Effect of early sowing	Effect of high plant density	IPM recommendation	Selected references
Clubroot	Early sowing can lead to warm, moist soils favouring infection	Dense plantings may exacerbate root stress and disease	Delay sowing to avoid warm wet conditions; maintain low to moderate densities; apply liming and sanitation	31, 32, 68
Light leaf spot	Increases autumn leaf infection and early epidemic development	Promotes moisture retention and disease spread	Use delayed sowing and lower densities in high‐risk regions	69, 70
Phoma stem canker	Varied – increases exposure to early infection in autumn unless plants are well‐established before primary inoculum is released	Varied – favors humid canopy and higher infection risk but can promote disease escape if lower leaves are shed before the pathogen grows to the stem	Many locations now sow early to establish large plants before primary inoculum arrives; use moderate density; combine with resistant cultivars	53, 71, 72
Powdery mildew	Less affected by sowing date; thrives in dense, humid canopies	Significantly increased by high density	Use wider row spacing and moderate densities	73, 74
Sclerotinia stem rot	More biomass and earlier flowering increase petal fall risk	Enhances canopy humidity, lodging, and petal trapping	Use moderate plant density; avoid overly early sowing in high‐risk fields	19, 75
Alternaria leaf spot	May increase duration of leaf wetness and lesion development	Dense stands restrict air flow, increasing humidity	Optimize spacing; improve canopy aeration	76
Verticillium stem striping disease	Early, dense stands may increase root contact and competition	Dense canopy may favour stress‐induced wilt	Avoid high plant density; select tolerant cultivars; maintain balanced sowing dates	48

### 3.4 Soil fertility and plant nutrients

Plants of different cultivars and grown in different conditions may vary in their composition, which may in turn affect the composition of pathogens establishing and feeding on those plants.^77^ Commercial fertilizers enhance crop productivity but can also influence plant susceptibility to pathogens. Key soil parameters including pH, the form of nitrogen (N), and the availability of essential macronutrients such as calcium (Ca), potassium (K), and phosphorus (P), as well as micronutrients like boron (B), magnesium (Mg), and zinc (Zn) have been shown to affect the development and severity of soil‐borne diseases.^78^ Among these, N fertilization plays a particularly complex role in modulating plant–pathogen interactions. Excessive N inputs are frequently associated with increased susceptibility to several foliar and stem diseases, notably sclerotinia stem rot and phoma stem canker. High N availability promotes vigorous vegetative growth and the formation of dense canopies, creating humid microclimates that favour the germination of sclerotia and enhancing conditions for infection and disease proliferation. Calcium nutrition has been shown to play a protective role against specific pathogens. Huber and Haneklaus^79^ demonstrated that Ca supplementation can suppress diseases caused by various *Sclerotinia* species by enhancing the structural integrity of the middle lamella, cell walls, and membranes. Additionally, Ca may directly inhibit the development of *Sclerotinia sclerotiorum* by reinforcing cell walls or binding with oxalic acid, which is a key virulence factor of the pathogen.^80^ In contrast, the application of P has been reported to increase the severity of *Sclerotinia* infections in several horticultural crops, emphasizing the importance of balanced nutrient management in disease control strategies.^80^ Campbell and Arthur^81^ also noted that Mg decreases clubroot severity in cabbage and broccoli. Conversely, soils low in Mg have been correlated with a higher incidence of clubroot disease.^82^ Further research has indicated that low N levels in the soil reduces clubroot symptoms in crucifers, while high N content exacerbates them. Additionally, B has been demonstrated to control clubroot in cabbage.^83^ However, the main method for clubroot management, has been liming, which has a direct effect by elevating soil pH and has been shown to improve Ca uptake and reduce disease outbreaks in crucifers, including OSR and vegetable Brassicas.^33^, ^84^ The effectiveness of lime depends on the lime type, application timing, and soil type.^15^, ^17^ Calcium cyanamide (CaCN_2_) was found to be an effective method for managing clubroot in cruciferous crops^85^ but the use of CaCN_2_ is about to be prohibited due to environmental concerns. An alternative strategy for mitigating the impact of sclerotinia stem rot involves applying biochar made from sewage sludge, which was found to reduce growth of certain fungi in soil, including *Sclerotinia*.^86^ Additionally, amending soil with a 2% sewage‐sludge humic acid (S–H) mixture reduced *Sclerotinia sclerotiorum* sclerotia viability below 20%.^87^ Furthermore, urea application (≥ 50 kg ha^−1^) generated soil volatiles that suppressed apothecia production from sclerotia.^88^ However, applying the S–H mixture as a soil amendment may exert adverse effects due to contamination by heavy metals, human pathogens, and other contaminants that pose risks to soil and water quality. Additionally, while humic acids can improve soil structure and nutrient availability, the agricultural efficacy of S–H mixtures remains inconsistent due to variability in sewage sludge composition. Source‐dependent differences in organic and inorganic constituents can significantly alter their performance as soil amendments.

### 3.5 Trap cropping (bait crops)

Trap cropping or using bait crops, a component of IPM, involves planting a secondary crop to attract pathogens away from the primary crop, thereby reducing pathogen pressure and associated disease risks. In OSR, this strategy has been used primarily for managing insect pests, which are vectors for several viral diseases.^89^ However, its direct impact on disease suppression is complex and context‐dependent. For example, the persistent viability of *Plasmodiophora brassicae* resting spores has led researchers to explore ways to trigger spore germination without a host as a potential strategy for clubroot control.^90^ Root exudates from non‐host plant species, such as leek (*Allium porrum*), winter rye (*Secale cereale*), or perennial ryegrass (*Lolium perenne*), have been shown to induce *Plasmodiophora brassicae* spore germination in nutrient solution.^91^ However, subsequent glasshouse and field trials failed to demonstrate short‐term soil sanitization effects.^91^ More recently, glasshouse studies have demonstrated the potential of non‐host species such as black‐grass (*Alopecurus myosuroides*), phacelia (*Phacelia tanacetifolia*), field poppy (*Papaver rhoeas*), and field pea (*Pisum sativum*) as bait crops to reduce the number of resting spores in clubroot‐infected soils.^22^ Similarly, alternative plants have been investigated to induce germination of sclerotia of *Sclerotinia sclerotiorum*, which has over 400 known host plant species.^92^ Recent studies found several plants, including dog poison (*Aethusa cynapium*), common orache (*Atriplex patula*), goat weed (*Hypericum perforatum*), match weed (*Matricaria recutita*), field pea (*Pisum sativum*), common sorrel (*Rumex acetosella*), or carnation clover (*Trifolium incarnatum*), induced germination but completely inhibited formation of new sclerotia. These findings indicate that while sclerotia may germinate in the presence of these non‐host crops, subsequent infection is minimal or absent, effectively interrupting the cycle of new sclerotia formation.^44^ Alternatively, *Pyrenopeziza, Alternaria* and *Leptosphaeria* species are primarily residue‐borne pathogens with rain and wind‐dispersed spores. Trap crops in the form of cover crops over OSR debris, may provide limited benefit by acting as a physical barrier to intercept or filter spores to reduce dispersal. However, they may also improve microclimatic conditions to favour maturation of fruiting bodies and spore dispersal. Further work is needed to investigate whether trap crops could break the dormancy of microsclerotia of *Verticillium longisporum*. Even if this is unlikely, the enhancement of soil microbial diversity and invertebrate activity facilitated by some plant communities may contribute by indirectly suppressing the pathogen.^93^

## 4 HOST RESISTANCE AND BREEDING STRATEGIES

Host–plant resistance is widely regarded as one of the most cost‐effective and environmentally sustainable strategies of IPM in WOSR in Europe.^11^, ^33^, ^94^, ^95^, ^96^, ^97^ In the context of breeding for disease resistance, it is common to distinguish between qualitative (major gene, R‐gene) and quantitative (polygenic, quantitative trait locus (QTL)‐based) resistance.^97^, ^98^, ^99^, ^100^, ^101^, ^102^ Qualitative resistance often provides a high level of protection but tends to be less durable due to pathogen adaptation; in contrast, quantitative resistance offers partial but more durable protection and is increasingly valued in modern breeding.^103^, ^104^, ^105^ Table 5 shows the current resistance status, associated limitations, and existing knowledge gaps for the major fungal, oomycete, and protist diseases affecting WOSR in Europe.

**Table 5:** Summary of the current resistance status, associated limitations, and existing knowledge gaps for major fungal, oomycete, and protist diseases affecting winter oilseed rape in Europe

Pathogen	Resistance status	Resistance durability	Limitations/gaps	Selected references
*Pythium* spp.	Very limited/negligible	Very low (no major resistant cultivars)	No resistant cultivars; screening protocols not standardized	
*Rhizoctonia solani*				
*Plasmodiophora brassicae*	Major R genes (e.g., Mendel‐derived) + emerging quantitative trait loci (QTLs)	Moderate (race‐specific resistance is effective)	Resistance overcoming due to virulent pathotypes; limited genetic diversity in commercial germplasm	33, 56, 57, 106, 107, 108
*Pyrenopeziza brassicae*	Various QTL and two known major genes	Unknown	Limited understanding of resistance mechanisms, limited research due to issue mainly in northwest Europe only.	109, 110
*Leptosphaeria maculans, Leptosphaeria biglobosa*	Major genes (mainly RLm4 and 7, RLmS and LepR1) plus quantitative resistance. RLm1, 2, 4 and 9 are not effective	Moderate: major‐gene resistances have been overcome by pathogen shifts; durability depends on management	Largely a success story but mutations arise constantly conferring newly virulent isolates	52, 111
*Erysiphe cruciferarum*	Unknown. Host age known to affect incubation period	Unknown	Broad host range of Brassicas likely to host a diverse pathogen population; resistance rare in *Brassica napus*; requires introgression from other *Brassica* species	43, 112
*Sclerotinia sclerotiorum*	Mainly promotion of disease escape (thicker cuticles, reduced flowering) and some QTL for tissue resistance	Low: full resistance not yet available; moderate durability expected for partial resistance	Oxalate oxidase expression detoxifies the main pathogenicity factor. Some inoculation methods are too invasive. Some ‘resistance’ actually escape due to flowering when inoculum was not present.	113, 114
*Alternaria brassicae*	Identified *Brassica napus* accession ‘ZY006’ as moderately resistant but some other species were highly resistant	unknown	Broad host range of Brassicas likely to host a diverse pathogen population	115
*Verticillium longisporum*	Quantitative disease resistance traits identified	Low–moderate: partial resistance only	Likely to be exacerbated by climate change	102

Breeding for resistance in WOSR faces several specific challenges: the polyploid nature of *Brassica napus* (AACC genome), frequent overcoming of major R‐genes by pathogen evolutionary shifts, limited availability of high‐resistance germplasm for many pathogens,^33^, ^116^ and environmental interactions (e.g., temperature, inoculum‐pressure) that affect expression of resistance (e.g., for phoma stem canker).^117^ Therefore, the shift in breeding strategy must be towards quantitative resistance, marker/genomic assisted selection, broader germplasm sourcing and integrated deployment.^112^

## 5 MONITORING, FORECASTING, AND DECISION SUPPORT

Various approaches may be used to monitor and forecast plant diseases, ranging from traditional field surveys and visual assessments to advanced digital and sensor‐based technologies (Fig. 4). While satellite and unmanned aerial vehicle (UAV)‐based imaging can observe large areas, they can usually only detect visible symptoms and therefore require curative crop protection products to be used, while weather‐based forecasts and inoculum‐detection (spore traps) allow for protectant products to be applied before infection occurs (Fig. 4). Traditional field‐based observations can now be backed‐up by rapid diagnostic tests, which can even be made in the field. Monitoring systems typically track disease incidence, severity, and environmental conditions that influence pathogen development, while forecasting models integrate weather data, host phenology, and pathogen biology to predict infection risk.

**Figure 4:**
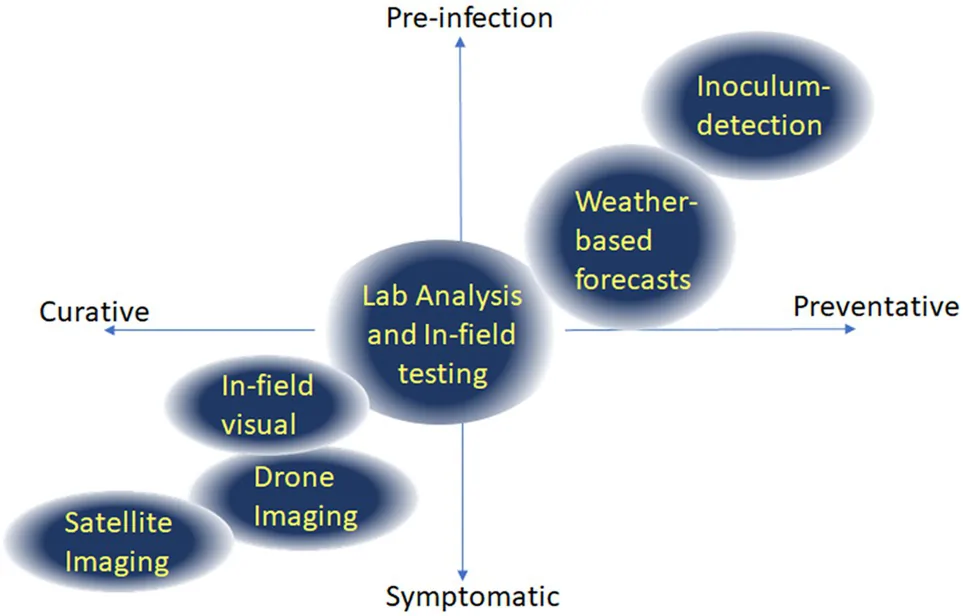
Monitoring and forecasting approaches used in integrated pest management (IPM) in relation to timing of visible symptom appearance and type of crop protection product, curative or preventative, that can be used as a result.

### 5.1 Imaging and in‐field visual assessments

In WOSR, there are relatively few practical examples of imaging by satellite or drones, either due to symptoms of most diseases appearing as discrete lesions that are often obscured by leaves above them and which for low resolution platforms, such as satellite imagery, would not be distinguishable, or for root and stem diseases, because symptoms appear after it is possible for any treatment to be effective. Mapping of root diseases such as clubroot is possible and allows identification of fields that require longer rotations to reduce the future disease.^118^ Optical phenotyping methods have been used but are often best applied to controlled conditions such as academic experiments or breeding plots.^119^ A system using UAV‐based thermal and RGB (red, green, blue) sensors, along with image registration demonstrated early detection of *Sclerotinia sclerotiorum* under controlled conditions, offering potential for field‐scale disease monitoring.^120^

Traditionally, in‐field assessments of plant visual appearance have been the main monitoring strategy for diseases. Observations generally begin during seed germination and emergence, which can be adversely affected by soil‐borne pathogens such as *Fusarium* spp., *Pythium* spp., and *Rhizoctonia* spp., and are primarily evaluated by the percentage of germinated seedlings relative to the number of seeds sown. Early seedling foliar diseases may be caused by *Hyaloperonospora parasitica*, the oomycete responsible for downy mildew. However, symptoms are rarely damaging and usually diminish in newly emerged leaves as the plant grows. *Leptosphaeria* spp., *Alternaria* spp. and *Pyrenopeziza* spp. are common pathogens on the rosette stage, and monitoring of leaf spot incidence relies mainly on estimating the proportion of leaf surface area affected. Disease severity is typically assessed using a 0–9 or 0–5 scale, depending on the required level of discrimination between genotypes. In the case of light leaf spot, disease assessments are conducted in the spring either in the field after a period of dry weather or by collecting and incubating leaves in a fridge to bring‐out symptoms (sporulation). Subsequent evaluations in late spring typically concern sclerotinia stem rot, which becomes evident after flowering and is usually scored by disease incidence (percentage of plants affected). This period also coincides with renewed *Alternaria* infections, which can affect leaves, stems and siliques – the latter representing the most economically damaging manifestation of the disease. In the preharvest phase, plants are also examined for symptoms of verticillium stem striping disease and phoma stem canker, the latter assessed by cross‐sectional area affected at the rosette leaf scars, but it is too late for control measures at this stage. However, late‐season disease assessments can be used to estimate yield.^27^, ^121^

### 5.2 In‐field diagnostic assays

In all cases, where visual symptoms are not easy to identify or are at very early stages of appearance, in‐field diagnostic tests are increasingly possible. Loop‐mediated isothermal amplification (LAMP), recombinase polymerase amplification (RPA) and other isothermal assays can be used with crude extracts in simple, portable devices to confirm the presence of a species or genotype. So far, LAMP assays have been developed for *Sclerotinia sclerotiorum*,^122^ a sub‐clade of *Leptosphaeria biglobosa*,^123^ for *Pyrenopeziza brassicae*,^124^ and for *Plasmodiophora brassicae*.^125^ Polymerase chain reaction (PCR)‐based assays are available for most other OSR pathogens, but will not be discussed here, as they remain largely laboratory‐based methods.

### 5.3 Weather based forecasting models and decision support schemes

Disease forecasting schemes for OSR have been developed to optimize fungicide applications and minimize unnecessary use within IPM programmes. Weather‐based forecasting models are advanced analytical tools used to predict the occurrence and development of crop diseases by integrating meteorological parameters. These models utilize data such as temperature, humidity, rainfall, and leaf wetness duration to assess the risk of pathogen infection. In the case of WOSR, such models are particularly useful for forecasting diseases like phoma stem canker,^126^ sclerotinia stem rot in Germany,^127^, ^128^, ^129^ and light leaf spot in the UK.^70^ By correlating weather conditions with disease epidemiology, these models prompt growers about when to be more vigilant to enable early detection or may even directly optimize the timing of fungicide applications. This approach reduces unnecessary chemical treatments, supporting both economic efficiency and environmental sustainability. Consequently, weather‐based forecasting models represent a crucial component of modern IPM systems in OSR cultivation. For example, the Rothamsted light leaf spot forecast was used for over 15 years and also provided crop‐specific information to guide growers on their choice of varietal resistance, fungicide programme and early or late sowing date.^34^, ^70^

Similarly, SkleroPro is a crop loss‐related forecasting model developed to predict sclerotinia stem rot in WOSR in Germany. Initially introduced by Koch *et al*.,^127^ the model estimates potential yield losses based on environmental conditions conducive to sclerotinia stem rot development. It utilizes weather data, crop rotation information, and economic factors to provide site‐specific recommendations for fungicide applications, with recent improvements to predict flowering stages^128^ and an improved sclerotia germination module,^23^, ^44^ which increased the model's predictive accuracy from 39% to 66%, with a sensitivity of 90%. A different approach to weather‐based forecasting of Sclerotinia is to predict the seasonal timing of spore release, which depends on soil temperature and moisture and the depth that sclerotia were buried^130^ and was used by Yong *et al*.^131^ to predict spore timing. Additionally, in France, the ‘RAISO‐Sclero’ decision support system predicts sclerotinia petal contamination in OSR and has been enhanced to use weather data to predict peak petal production and Sclerotinia spore availability.^129^

The Rothamsted LLS, Rothamsted Phoma, RAISO‐Sclero and SkleroPro models are examples of decision support systems for plant protection, based on meteorological data, which are relatively cost‐effective to obtain. Some of these have other inputs, such as the amount of disease the previous year and in all cases, they are assessed experimentally and compared with observations of disease progress in field crops or plots. Predictive models have been developed for stem canker, integrating knowledge on the release of ascospores of *Leptosphaeria maculans* and *Leptosphaeria biglobosa* obtained through microscopic and/or molecular monitoring.^27^, ^132^ Combining these approaches provides not only qualitative but also species‐level identification of phytopathogenic fungi, thereby improving the precision of timing and dosage of chemical control measures.

### 5.4 Inoculum detection

Although weather‐based forecasts can help to optimize crop protection, there can still be unnecessary interventions made if weather conditions suggest diseases will develop but inoculum is actually absent and therefore little, or no disease will result. There can also be variability in performance of weather‐based forecasts, which may work well in one location but not another. For example, the SkleroPro model for Sclerotinia, which works well in Germany, was found by Gladders *et al*.^133^ to be a poor indicator of sclerotinia incidence in the UK because different regions of the UK that had a similar frequency of infection conditions being met, often resulted in very different amounts of sclerotinia infection due to differences in inoculum availability.

In those cases, and particularly for sporadic diseases or those that may arrive on the wind from long distances, aerobiological monitoring using spore traps provides a means to detect imminent disease risk before infection has even taken place. This approach can also be enhanced by integration with weather‐based infection models. As such, it allows preventative applications of crop protection products at the most optimal time. Automated systems using DNA, immunological tests, or based on image recognition of spores are starting to be developed. For example, Bioscout uses image recognition of spores impacted onto a transparent tape and can provide same‐day information on ascospores of *Leptosphaeria maculans* (www.bioscout.com.au). Alternatively, even where spore trap samples are sent to a laboratory for testing, results can often be obtained before visible symptoms appear and in time for crop protection products to be effective. Monitoring of airborne ascospores has been implemented in numerous countries worldwide, with monitoring established in the UK for *Leptosphaeria maculans*,^126^, ^134^ light leaf spot^34^ and sclerotinia stem rot.^135^ In some cases, monitoring has provided data for simpler weather‐based models to be produced (see earlier).

In Poland, such monitoring has been conducted on a nationwide scale within the SPEC System for Forecasting Epidemics operating since 2004, covering all major OSR‐growing regions of the country,^136^ in some years supplemented with the detection of *Leptosphaeria* species and avirulence genes in *Leptosphaeria maculans*.^132^, ^137^ In addition to the UK and Poland, airborne spore monitoring for phoma leaf spot has also been used in Czech Republic and Lithuania.^138^, ^139^, ^140^, ^141^ It has also been used for *Pyrenopeziza brassicae* in Poland.^35^

For phoma stem canker, in addition to monitoring ascospores, which in many locations are regarded as the primary source of inoculum, rain‐splashed conidia (pycnidiospores) can be a significant source requiring monitoring, which would primarily require a DNA or immunological detection method.^142^, ^143^

Monitoring airborne spores can provide additional information in tracing the source of airborne spores using backward air mass trajectory analysis.^144^ This can be done on large or very local scales but source identification is particularly challenging in regions with multiple source locations, for example, where pathogen presence is widespread. Effects of the density of inoculum sources on crop exposure have also been modelled for phoma stem canker,^145^, ^146^ which can help to identify effective separation distances.

In addition to detecting individual species to improve crop protection, air sampling can also be used to monitor changes in pathogen populations if known genetic traits can be detected, such as those conferring fungicide resistance or pathotypes.^147^, ^148^, ^149^

Importantly, aerobiological monitoring data are valuable not only for agricultural purposes but also for public health. Many phytopathogenic fungi are known to cause allergic reactions in humans and animals. For instance, *Alternaria* spp. produce well‐characterized allergenic proteins in their spores,^150^ and allergenic proteins have recently been identified in *Leptosphaeria* spp.^151^ Hence, monitoring airborne fungal spore concentrations provides dual benefits, supporting IPM and contributing to the assessment of allergenic risk to humans and animals.

## 6 BIOLOGICAL CONTROL AGENTS AND MICROBIAL ANTAGONISTS

Biological control is currently not a key element of IPM strategies for OSR. Few products are registered, and even fewer are currently used by farmers. This is likely due to the relatively low impact of biological control agents (BCAs) on disease control compared to conventional fungicides and the investment required to apply biologicals to fields. WOSR is grown by medium‐ and large‐scale farms on very large areas, so the investment in biological control agents is very significant, given their prices.

Microbial bio‐pesticides can offer an alternative to fungicides.^152^ Many companies are ‘bioprospecting’, in particular screening the enormously diverse soil mycobiome for potential beneficial microbes.^77^ Biocontrol is usually not as effective as traditional fungicides, particularly for foliar applications but it is relatively efficient against soil‐borne pathogens and those surviving on crop debris and in soil. Indeed, true biologicals, rather than biopesticides, should survive for longer periods providing long‐term protection. A promising discovery with this potential was the identification of two endophytes (*Beauveria bassiana* and *Pseudogymnoascus pannorum*) that each reduce the severity of stem canker.^153^

For *Sclerotinia* control, the most well‐known and most frequently used product is Contans WG, which contains the coelomycete fungus *Coniothyrium minitans*, later renamed to *Paraconiothyrium minitans* and renamed again to *Paraphaeosphaeria minitans*.^154^ It is a superparasite of sclerotia formed by fungi belonging to the *Sclerotinia* genus, including *Sclerotinia sclerotiorum*, and is distributed globally as a soil microbe.^155^ It was experimentally proved that *Paraphaeosphaeria minitans* reduces sclerotial survival and apothecial production of *Sclerotinia sclerotiorum* in field‐grown OSR,^156^ although later studies showed contradictory results, some studies demonstrated the usefulness of aerial application of *Paraphaeosphaeria minitans*, whereas other studies found neither an effect of Contans WG on petal infection by *Sclerotinia* ascospores nor a difference in the germination rate of apothecia between Contans‐treated and untreated fields.^157^ Ongoing research to find new biocontrol agents for *Sclerotinia* spp. has used high resolution scanning electron microscopy (to visualize damage to sclerotia), and gas chromatography–mass spectrometry (GC–MS) analyses of antifungal compounds tested *in vitro*.^158^

An interesting recent approach was to combine the use of synthetic fungicides with *Coniothyrium  minitans* or *Trichoderma asperellum*, which reduced infection substantially compared to untreated control field plots.^159^ The adoption of such a strategy is in line with IPM guidelines and shows a direction towards integrated plant protection strategies that maintain crop health without adverse effects on the environment.^160^ Similar effects were previously shown by Hu *et al*.^161^ who used an unknown species of *Trichoderma* with carbendazim fungicide, which is currently banned, due to operator safety concerns.^162^ Numerous species in the genus *Trichoderma* are commonly used to reduce the adverse effect of many plant pathogens.^163^ To control sclerotinia stem rot, *Trichoderma harzianum* proved to have strong mycoparasitic abilities including strong activity of cell wall degrading enzymes against the sclerotia of *Sclerotinia sclerotiorum*.^164^ In addition, some other fungi pathogenic to *Sclerotinia sclerotiorum* have been described, such as *Sporidesmium sclerotivorum*,^165^ renamed to *Teratosperma sclerotivorum* and *Gliocladium virens*.^166^ The latter fungus was less effective as compared to *Coniothyrium minitans* due to its lower activity at a lower temperature range^166^ but it may become increasingly useful due to global warming. The bacterium *Bacillus amyloliquefaciens* has also been effective against some diseases of WOSR when applied in the form of the bicinchoninic acid (BCA) preparation Serenade.^167^

Recently, Auer *et al*.^168^ found that *Acremonium alternatum* reduced clubroot symptoms in some, but not all susceptible cultivars inoculated with an aggressive *Plasmodiophora brassicae* isolate at moderate (10^6^ spores mL^−1^) and high (10^7^ spores mL^−1^) concentrations. Moreover *Acremonium alternatum* enhanced plant vigour, leading the authors to conclude that *Acremonium alternatum* holds some promise against *Plasmodiophora brassicae*, especially when combined with resistant cultivars. It is also strongly recommended to use cultural practices aimed at reducing sclerotia number (of *Sclerotinia* but similarly for any inoculum of soilborne diseases) in the soil.^19^ In the case of biocontrol agents one of the biggest challenges is achieving a long shelf life for the formulation.^169^

## 7 CHEMICAL CONTROL AND FUNGICIDE USE

It is important to apply fungicides before diseases becomes severe. For diseases with long symptomless incubation periods such as light leaf spot, forecasting tools are important to guide growers as to the correct strategy. Most currently recommended fungicide products contain azoles (demethylation inhibitors (DMIs), FRAC code #3), mixed or alternated with other modes of action such as succinate dehydrogenase inhibitors (SDHIs, FRAC code #7) or strobilurins (quinone outside inhibitors (QoIs), FRAC code #11). These are best applied ahead of significant disease development.^45^, ^170^ Methyl benzimidazole carbamates (MBCs, FRAC code #1) are no longer applied due to legislation banning them, and because fungicide resistance is already widespread in multiple pathogens, for example, *Sclerotinia sclerotiorum* and *Pyrenopeziza brassicae*.^171^, ^172^

For light leaf spot and phoma leaf spot, a mid‐autumn application is suggested in regions prone to high levels of disease and this is often before visible symptoms of light leaf spot occurs, although backward crops may suffer a yield reduction if autumn‐applied fungicides have a growth regulatory effect. In addition, a spray at the start of stem extension is recommended against light leaf spot if the disease incidence is over 15% and the final efficacy of the fungicide spray may be modulated by the availability of sulphur.^173^ The disease is hard to see following wet weather, except for later, advanced symptoms of leaf bleaching and distortion. Disease assessment can be improved by incubating collected leaves in a closed plastic bag in a cool room (ideally 5–10 °C) for 3–4 days before looking for sporulation, which should then be visible.

For phoma stem canker, fungicides have little or no effect once the fungus has reached the stem, indeed, the best timing of fungicide application is after the first significant release of ascospores in the autumn, ideally before more than 10–20% of plants have phoma leaf spots. The timing of spore release varies each year due to the weather with wet summers promoting an early release (10% of plants with phoma leaf spots by late September). It is advised that on susceptible varieties a two‐spray autumn fungicide regime is best if phoma leaf spotting occurs early in the autumn (before mid‐October), while a single autumn spray is effective if phoma leaf spotting is late (appearing after mid‐October). On more resistant varieties, fungicide applications can be reduced or omitted completely (against phoma stem canker) if phoma leaf spotting is late.^134^

*Sclerotinia* spores can only infect healthy green tissue if the spores are in contact with a fallen petal or senesced leaf, which act as an undefended substrate and provides enough resource for the pathogen to secrete oxalic acid onto the leaf or stem it is on, killing a patch of plant tissue.^114^ The fungus then grows into the killed tissue and continues to secrete more oxalic acid. Infections can be prevented by foliar fungicides applied before the main flowering period. Generally, most available fungicides provide some protection if applied at the correct time.^174^ Currently a mix of SDHI and DMI (e.g., bixafen and prothioconazole), or a mixture of QoI and SDHI (e.g., dimoxystrobin and boscalid) fungicides are reported to be the most effective, although some straight DMIs such as prothioconazole alone are also effective. For a single spray application, the optimal time is around growth stage (GS) 6.3 – roughly a third of the way into flowering, based on flower production on the main stem. Fungicides are thought to provide protection for 3 to 4 weeks but some products may provide limited activity for longer. However, at high‐risk locations, a second application towards the end of flowering may be necessary to protect the whole of the flowering period. Foliar applications of B were found to reduce *Sclerotinia* infection substantially – these were not for crop nutrition (fertilizer) purposes but were found to prime resistance responses and possibly had an additional direct effect.^175^

*Verticillium longisporum* is difficult to control by fungicides as it is soil borne and so the disease is most effectively controlled by other means such as lengthening rotations and using resistant varieties. Similarly, powdery mildew and Alternaria leaf spot, although affecting plant vigour are usually not the target of fungicide spray decisions in Europe because they have a lower impact on yield compared to clubroot, phoma stem canker, light leaf spot and sclerotinia stem rot.

Despite advice to use fungicides mixed as a combination of at least two modes of action and preferably alternating effective modes of action, a loss of active ingredients has increased selection pressure on pathogens to reduce sensitivity to the remaining available fungicides. As a result, there has been a gradual erosion of efficacy of some key fungicides, including DMIs (FRAC code #3). For example, in 2024, a new mutation was found in populations of *Leptosphaeria biglobosa* conferring about a seven‐fold reduction in sensitivity to DMI fungicides.^176^ Similarly in *Leptosphaeria maculans*, a 3‐10‐fold decrease in sensitivity to DMI fungicides was found to be caused by a promoter insert, causing over‐expression of the CYP51 gene^177^ and other factors reducing sensitivity have been identified.^178^ Fortunately, no decrease in sensitivity to QoIs (FRAC code #11) or SDHIs (FRAC code #7) has been found so far in Europe in *Leptosphaeria maculans*. In contrast, reduced sensitivity to QoI's was found in *Sclerotinia* spp. in Canada^120^ and reduced sensitivity to DMIs was also found in *Sclerotinia* spp. in the United States.^179^

*Pyrenopeziza brassicae* has also become less sensitive to DMI fungicides in recent years in Europe compared to reference isolates collected in the mid‐1990s and 2000s. This is mainly due to target‐site alterations such as G460S or S508T, which typically are also associated with promoter inserts, collectively causing 9–50‐fold reduced sensitivity compared to reference isolates.^180^ Widespread resistance to MBC fungicides in *Pyrenopeziza brassicae* was already found by Carter *et al*.^172^ over 10 years ago.

From a ‘One‐Health’ perspective, the use of fungicide modes of action in agriculture that are the same as those used in human medicine gives potential for cross‐over of resistance mutations selected for in the agriculture environment into the clinical setting and so efforts are needed in both clinical and agricultural settings to enhance monitoring for fungicide resistance and to reduce selection and proliferation of fungicide‐resistant individuals.^181^, ^182^

## 8 CHALLENGES TO CURRENT IPM IMPLEMENTATION

Each year brings new advances in IPM strategies to control pests in WOSR.^12^ However, much is still unknown or under revision about the pathogens themselves, including their classification, which may not be standardized between countries. This can bring problems, such as the use of different pathotyping systems in *Plasmodiophora brassicae* for monitoring of clubroot.^101^ In *Leptosphaeria maculans*, the previously postulated single gene‐for‐gene relationship appeared to be complicated, with more plant genes conferring resistance to the same variant of the pathogen or epistatic effects of some avirulence‐resistance gene interactions.^183^ New races or lineages of the pathogens are discovered and races that were previously very rare may emerge to become predominant. For example, Carmody *et al*.^184^ found a new lineage of *Pyrenopeziza brassicae*, causing a new disease on Brassicas, called chlorotic leaf spot in north‐western United States, which so far has not reached Europe, and King *et al*.^185^ found the ‘canadensis’ subclade of *Leptosphaeria biglobosa* in Europe for the first time in 2022 on wasabi plants but the isolates were able to infect WOSR. It is not yet known whether this subclade has started to spread onto WOSR in Europe. In addition to changes in virulence of pathogens, there is also potentially rapid changes to pathogen populations with respect to the loss of susceptibility to some fungicide modes of action.^177^ The diseases are subjected to the boom and boost cycles which also makes disease control difficult.^186^

## 9 FUTURE CHALENGES, PERSPECTIVES AND INNOVATIONS

Digital agriculture (remote sensing) and precision IPM or precision agriculture, integrates advanced data analytics with targeted resource application to optimize crop productivity while reducing environmental impact and is particularly good for irrigation scheduling and spatially‐variable fertilizer application.^187^, ^188^ Remote sensing plays an important role in precision agriculture, particularly for high‐value crops by providing high spatial, spectral and temporal resolution data essential for disease detection.^189^, ^190^ UAVs and ground‐based (fixed or tractor‐mounted) platforms offer higher‐resolution down to < 1 mm^2^ and avoid problems of cloud cover but do not offer such cost‐effective coverage of large areas compared to satellites, which have poorer resolution (typically 1–400 m^2^) and may not allow for a full range of spectral indices if only RGB and near‐infrared (NIR) bands are available.^191^ Therefore, for early detection, necessary for effective control, proximal platforms appear to be the best solution, but even then, issues of shadow, leaf angle, surface wetness, obscuring by younger leaves and transmitted sunlight, pose challenges for field use. Thermal wavebands and fluorescence imaging may offer additional solutions^120^, ^189^ as may the combining of imaging with chemometric and machine learning approaches, which enhanced the accuracy of sclerotinia stem rot detection in OSR leaves.^192^ Recent advancements in artificial intelligence (AI)‐based image processing may lead to simplified, user‐friendly and robust systems for real‐time practical implementation. A future possibility combining imaging with biotechnology (next subsection) could be the use of ‘sentinel’ plants, which have been genetically engineered to fluoresce when they become infected by a pathogen.^193^ Since then, some companies have received investment to create crops that will have a small proportion of them acting as living biosensors by fluorescing under certain stresses, including pathogen attack, for example, InnerPlant (innerplant.com). At the time of writing, no peer‐reviewed scientific publications have reported practical use of this technique in field conditions. It would be important for such engineered plants to be insensitive to opportunistic or ‘attempted’ infections caused when spores may land on the wrong host and attempt to infect. Otherwise, the sentinel plants would likely fluoresce constantly, which would limit their utility.

### 9.1 Other approaches

In‐field diagnostics to allow early identification of incubating infections or captured spore trap samples, are likely to continue to improve, be linked to wireless reporting and become cheaper. Plant breeding to improve resistance is also now aided by use of molecular markers, genomics and potentially in the future, through gene editing.

### 9.2 RNA‐based technologies and biotechnological tools

Epigenetic control of effector gene expression has been studied in *Leptosphaeria maculans* and is in progress for a number of other pathogens of OSR.^194^ Knowledge of pathogen effectors and other essential genes involved in infection identifies potential target genes to silence as a way to inhibit infection using RNA interference (RNAi).^195^ The method can be applied to any pathogen and uses double‐stranded RNA molecules, which are designed to target specific genes, by blocking translation of messenger RNA (mRNA) into proteins resulting in reduced growth or decreased virulence of the targeted pathogen. This can be done by spraying artificially‐produced RNA onto plants to cause spray‐induced gene silencing (SIGS) or a genetic modification to the host can be made to express the designed RNA, which is called host‐induced gene silencing (HIGS). Recently, RNAi‐mediated targeting of monooxygenase SsMNO1 was developed to control sclerotinia stem rot caused by *Sclerotinia sclerotiorum*.^196^ The technology can also be used to control insects; for example, SIGS was used to reduce the population of pollen beetles.^197^ This technology offers a highly specific and environmentally friendly alternative to conventional chemical control in the case of SIGS and to augment resistance breeding in the case of HIGS. Furthermore, genome‐editing tools such as CRISPR/Cas systems are being applied to enhance OSR resistance by modifying susceptibility genes or introducing more durable resistance traits.^198^ Synthetic biology enables the design of novel defence‐related peptides and regulatory RNAs to strengthen plant immunity. Advances in transcriptomic analysis help identify key regulatory RNAs involved in plant–pathogen interactions, facilitating targeted biotechnological interventions. A good example is the comparative transcriptome analysis of canola carrying a single *versus* stacked resistance genes against clubroot.^199^ The integration of these RNA‐based technologies with traditional breeding programmes is accelerating the development of resistant cultivars. Overall, RNA‐driven biotechnology offers sustainable, precise, and adaptive solutions for managing WOSR‐related diseases.

### 9.3 Climate change

The climate of Europe is predicted to become warmer compared to the baseline of 1960–1990 and there is strong evidence that this has already occurred with five of the ten hottest summers in Europe being in the last 20 years. Winters are predicted to be warmer and wetter, while summers are predicted to be much hotter and drier, particularly in southern Europe,^200^ which helps pathogens to continue their development in plants of WOSR.^72^

There will also be more extreme weather events. These changes will affect both disease development by causing pathogens to release spores earlier in the year for spring infections or later for autumn infections. Crop development may also be advanced and so it is difficult to predict whether there will be significant changes. It does appear that warmer conditions can increase the impact on crop yield, particularly of root and stem‐infecting pathogens such as *Plasmodiophora brassicae*, *Leptosphaeria maculans* and *Verticillium longisporum* because the plant will face increased water‐stress as a result of heat and drought, which will exacerbate effects of any disease disrupting water supply to upper parts of the plant.^201^, ^202^ Moreover, higher humidity and warmth accelerated the maturation of fruiting bodies and the release of infectious spores, as it is the case reported in *Leptosphaeria* spp.^203^, ^204^

Certainly, under warmer conditions, assuming sufficient moisture, polycyclic disease will have more cycles of infection so powdery mildew, light leaf spot (during winter), and Alternaria may increase in severity.^16^ However, other changes to leaf wetness durations could reduce disease severity in some cases.^205^, ^206^ Furthermore, ‘indirect’ changes are likely due to changes in agricultural practices, and other crops grown in response to climate change. For example, *Brassica juncea*, which is known to be more drought tolerant may be grown in some areas where *Brassica napus* is currently grown and will have a different disease profile. Although climate change is a gradual phenomenon, it can take many years to develop new varieties that are resistant to diseases that may become more serious in the future. High temperature shocks may also render host qualitative resistance genes as dysfunctional at least temporarily and sustained high temperatures may also reduce quantitative resistance.^117^, ^207^ It may be possible to introduce both climate and disease resistance traits from synthetic crosses of wild ancestral species but it will likely require constant vigilance for newly arising virulence.^208^, ^209^

## 10 CONCLUSION

IPM for WOSR in Europe has advanced considerably over the past few decades, yet significant challenges remain. This review highlights that sustainable disease management relies on combining cultural practices, host resistance, and precise fungicide use, supported increasingly by decision support systems, remote sensing, and predictive modelling. Key findings show that while progress has been made in understanding pathogen biology, population structure, and epidemiology, the complexity and diversity of WOSR pathogens continue to limit the effectiveness of single‐strategy approaches. Furthermore, increasing pressure from insect pests, combined with the ongoing loss of chemical active substances in the European Union (EU) and gain of resistance to many active ingredients, underscores the need for more diversified and ecologically based IPM strategies, including longer or more diverse rotations.

While biological control agents offer promise, their field efficacy in WOSR remains limited and inconsistent. Overall, sustainable disease management will require a balanced, flexible approach that integrates traditional agronomic practices with emerging biological and digital innovations to maintain resilient WOSR production in Europe.

## CONFLICT OF INTEREST

The authors declare no conflict of interest.

## Data Availability

Data sharing not applicable to this article as no datasets were generated or analysed during the current study.
